# Deciphering the Role of Filamin B Calponin-Homology Domain in Causing the Larsen Syndrome, Boomerang Dysplasia, and Atelosteogenesis Type I Spectrum Disorders via a Computational Approach

**DOI:** 10.3390/molecules25235543

**Published:** 2020-11-26

**Authors:** Udhaya Kumar S., Srivarshini Sankar, Salma Younes, Thirumal Kumar D., Muneera Naseer Ahmad, Sarah Samer Okashah, Balu Kamaraj, Abeer Mohammed Al-Subaie, George Priya Doss C., Hatem Zayed

**Affiliations:** 1 School of Biosciences and Technology, Vellore Institute of Technology, Vellore, Tamil Nadu 632014, India; s.udhayakumar2018@vitstudent.ac.in (U.K.S.); srivarshini307@gmail.com (S.S.); thirumalkumar.d@gmail.com (T.K.D.); 2 Department of Biomedical Sciences, College of Health and Sciences, Qatar University, QU Health, Doha 2713, Qatar; sy1203986@student.qu.edu.qa (S.Y.); ma1403939@student.qu.edu.qa (M.N.A.); so1404563@student.qu.edu.qa (S.S.O.); 3 Department of Neuroscience Technology, College of Applied Medical Sciences in Jubail, Imam Abdulrahman Bin Faisal University, Jubail 35816, Saudi Arabia; bkranganayaki@iau.edu.sa; 4 Department of Clinical Laboratory Sciences, College of Applied Medical Sciences, Imam Abdulrahman Bin Faisal University, Dammam 31441, Saudi Arabia; amnalsubaie@iau.edu.sa

**Keywords:** FLNB, CH2 domain, Larsen syndrome, boomerang dysplasia, atelosteogenesis type I, molecular dynamics simulation (MDS)

## Abstract

Filamins (FLN) are a family of actin-binding proteins involved in regulating the cytoskeleton and signaling phenomenon by developing a network with F-actin and FLN-binding partners. The FLN family comprises three conserved isoforms in mammals: FLNA, FLNB, and FLNC. FLNB is a multidomain monomer protein with domains containing an actin-binding N-terminal domain (ABD 1–242), encompassing two calponin-homology domains (assigned CH1 and CH2). Primary variants in FLNB mostly occur in the domain (CH2) and surrounding the hinge-1 region. The four autosomal dominant disorders that are associated with *FLNB* variants are Larsen syndrome, atelosteogenesis type I (AOI), atelosteogenesis type III (AOIII), and boomerang dysplasia (BD). Despite the intense clustering of *FLNB* variants contributing to the LS-AO-BD disorders, the genotype-phenotype correlation is still enigmatic. In silico prediction tools and molecular dynamics simulation (MDS) approaches have offered the potential for variant classification and pathogenicity predictions. We retrieved 285 FLNB missense variants from the UniProt, ClinVar, and HGMD databases in the current study. Of these, five and 39 variants were located in the CH1 and CH2 domains, respectively. These variants were subjected to various pathogenicity and stability prediction tools, evolutionary and conservation analyses, and biophysical and physicochemical properties analyses. Molecular dynamics simulation (MDS) was performed on the three candidate variants in the CH2 domain (W148R, F161C, and L171R) that were predicted to be the most pathogenic. The MDS analysis results showed that these three variants are highly compact compared to the native protein, suggesting that they could affect the protein on the structural and functional levels. The computational approach demonstrates the differences between the FLNB mutants and the wild type in a structural and functional context. Our findings expand our knowledge on the genotype-phenotype correlation in FLNB-related LS-AO-BD disorders on the molecular level, which may pave the way for optimizing drug therapy by integrating precision medicine.

## 1. Introduction

Human Filamins constitute a group of large actin-binding proteins comprised of 24 immunoglobulin-like repeats and actin-binding domain (ABD), forming a dynamic structure via crosslinking cytoskeleton filaments. Filaments cooperate with various signaling proteins in the cytosol and transmembrane receptors through which they connect the cell membrane to the cytoskeleton mechanically and functionally [1]. In addition, filaments play a role in skeletal development through intracellular signaling pathways [1,2]. In mammals, the filamin family encompasses three main homologous proteins: filamin A (FLNA), filamin B (FLNB), and filamin C (FLNC). The first discovered member of the family, FLNA, was considered the most abundant and widely distributed member of this lineage [3,4]. FLNB and FLNC were subsequently identified. Polymorphisms in the *FLN* genes result in abnormal expression of filamin proteins, causing a broad range of congenital abnormalities. FLNA and FLNC polymorphisms cause diseases involving the cardiovascular, nervous, and skeletal systems [1,5,6,7]. However, FLNB polymorphisms are uniquely involved in skeletal conditions, signifying the critical role of FLNB protein in developing the skeletal system [8]. FLNB was cloned in 1998 [9]; it is confined at the short arm of chromosome 3 (3p14.3) [10], and it codes for a 2602 amino acid-protein, organized into an ABD constituted by a C-terminal dimerization domain including two calponin-homology (assigned CH1 and CH2) domains. A central rod-like domain includes 24 repeats interrupted by hinge-1, which connects repeats 15 and 16, and hinge-2, which connects repeats 23 and 24 [11]. FLNB acts as a scaffold that cooperates with different channels, GPIbα [6], receptors, presenilin [12], transcription factors, and signaling molecules [13].

*FLNB*-related disorders include skeletal malformations involving skeletogenesis, irregular joint formation, and vertebral segmentation [14]. Five primary *FLNB*-related skeletal disorders have been described and classified based on their clinical manifestations and genetic etiology into two broad groups. Homozygosity and/or compound is associated with spondylocarpotarsal synostosis (SCT, OMIM 272460), a recessive condition that features fused vertebrae, fused carpal, and tarsal joints [2,7,15,16]. The second group encompasses a range of autosomal dominant FLNB-related disorders caused by genetic variants, such as small in-frame insertions or deletions and missense variants. This group includes severe disorders in which bones are under modeled or do not initiate ossification, e.g., atelosteogenesis type I (AO-I, OMIM 108720), boomerang dysplasia (BD, OMIM 112310), and atelosteogenesis type III (AO-III, OMIM 108721). It also includes the mildest phenotype, described as Larsen syndrome (LS, OMIM 150250), that constitutes massive joint dislocations and spinal deformity [17,18].

The mechanism by which missense variants in *FLNB* cause skeletal diseases remains unclear. However, variants in the *FLNB* gene predominantly occur in the domain (CH2) and near the hinge-1 region [19,20]. This non-random localization suggests that these domains play a crucial role in interacting with other molecules and maintaining protein conformation [21,22]. Most of the variants in FLNB occur only in domain CH2 and not CH1, suggesting a significant role of the CH2 domain in normal skeletogenesis and protein function [14,17]. There is growing evidence that the mechanism underlying the recessively inherited phenotype SCT is distinct from that in the autosomal dominant range of disorders that includes LS-AO-BD phenotypes in several ways. First of all, disorders due to amino acid substitutions in the CH2 and first-rod domains are phenotypically different from SCT, resulting in a truncated protein, which leads to the loss of protein expression [15]. Second, heterozygous carriers with variants associated with SCT were shown to cause only negligible or no manifestations of skeletal abnormalities, implying that the LS-AO-BD disorders are unlikely to be due haploinsufficiency. These findings suggest that gain-of-function variants are linked to LS-AO-BD disorders. Nevertheless, the implications of increased actin-binding ability conferred by these variants on a cellular level remain poorly understood. During ABD binding to F-actins, CH1 plays a dominant role, while CH2 plays a regulatory/supportive role [22,23].

According to the Human Gene Mutation Database (HGMD), 93 variants have been identified in *FLNB* to date, of which the majority are missense variants [23], and very few are nonsense variants [14]. Missense variants may produce nonfunctional proteins. Thus, it is plausible that filamin–actin-binding may be modulated physiologically. The gain-of-function effect may impact the cellular mechano-transduction mechanism via increasing actin–filamin avidity [20]. Most of the variants reported in *FLNB* were associated with the LS phenotype, with several variants being highly recurrent as de novo in unrelated patients; these include variants in the CH2 domain, such as c.488A > C; Q163P, c.501C > A; D167Q, and c.500A > G; D167G [19], along with other variants in repeats around the flexible hinge region, such as c.4292T > G; L1431R in repeat 13 [24]. Additionally, several variants have been associated with the AOI/III and BD phenotypes, signifying that the abnormality in osteogenesis observed in these conditions may have its equivalent in patients with LS subclinically. In some instances, the same pathogenic variant was found to be associated with different phenotypes. For instance, c.502G > T (G168C) has been associated with LS, AOI, and AOIII [19,24,25], and c.604A > G (M202V) was found to be associated with both AOI and AOIII phenotypes [14], while others were found to be unique for one phenotype, such as c.442T > A; W148R, which was found to underly the AOI phenotype [25]. However, it remains poorly understood why variants in the same CH2 domain region are associated with different phenotypes. While few variants have been previously reported to be related to the BD phenotype (c.703T > C; S235P, c.512T > G; K171R [17] and c.605T > C; M202T [19], those that have been studied all occurred in the CH2 domain. The interfamilial variation in phenotypes was shown to vary widely among patients. For instance, the variant c.5071G > A (G1691S) was previously reported in six unrelated LS patients presenting with varying consequences, ranging from mild to severe cases [24]. Another case with this variant presenting with the AOIII phenotype was described, indicating phenotypic overlap [25]. The phenotypic relatedness between LS and AOIII is reinforced by survival reports in patients with AOIII [26]. Although there is evidence of the intense clustering of variants that cause LS-AO-BD conditions, the phenotype relationship remains unclear. Thus, understanding the nature of the missense variants altering these proteins will help find new drug targets for better treatment options for the LS-AO-BD spectrum of conditions.

This study presents a computational strategy to study missense variants in the *FLNB* gene and prioritize the most pathogenic variants to establish an exact genotype-phenotype relationship. In this study, information regarding the *FLNB* gene product’s biological function and the natural variants was obtained from UniProt. The germline variants underlying the CH2 domain were retrieved from the HGMD. Our previous study successfully established a computational approach to investigate the genetic variants’ impact on the protein’s function and structure [27,28,29,30,31,32,33,34,35]. Our previous study analyzed the structural deviations (transformation of bend to the coil) of G1691S variants responsible for Larsen syndrome [30]. We adapted in silico tools for evaluating the stability, pathogenicity, and conservation patterns due to missense variants. Finally, we conducted a molecular dynamics simulation (MDS) to study the structural changes after introducing the variants in an atomistic context. The MD trajectory analyses were performed for the whole native and mutant proteins and the CH1 and CH2 domains of native and mutants. Our findings may provide a practical understanding of FLNB variants on a structural and functional level. They may serve as a platform for the discovery of new drug targets in FLNB-related disorders.

## 2. Materials and Methods

### 2.1. Collection of Variants

Four literature databases were used to collect information related to variants: Google Scholar, PubMed, Science Direct, and PubMed Central. The search terms that were applied were broad (“*FLNB* variants” OR “Filamin B variants”) in order to collect all possible studies. 

The UniProt’s biological function (https://www.uniprot.org/uniprot/O75369) was used to collect information regarding the biological functions of the *FLNB* gene product and its natural variants [36,37,38]. The HGMD was used to obtain the germline variants in the *FLNB* gene underlying the disease [39] (Table S1).

The 3-D structure of FLNB was procured from the RCSB Protein Data Bank (PDB ID: 2WA5) [40]. Captured variants were assessed for their pathogenicity, stability, and conserved existence.

### 2.2. Evolutionary Conservation

Align-GVGD is a tool that resolves missense variants as enriched deleterious to enriched neutral via the usage of multiple protein sequence alignments and amino acid (AA) biophysical features [41]. ConSurf was utilized for assessing the evolutionary conservation of AA residues premised on the phylogenetic connection between all the homolog protein sequences of the FLNB [42,43].

### 2.3. Prediction of Pathogenicity

PredictSNP was used to predict the protein function arising from the amino acid substitution [44]. PredictSNP is combined and developed with eight prediction algorithms. MAPP [45] aims to predict the adverse impact of all potential substitutions of amino acids on the protein function. If present, the phenotypic influence of an nsSNP is predicted by nsSNPAnalyser [46] and evolutionary evaluation of the coding single nucleotide polymorphisms (SNPs) by PANTHER [47]. PhD-SNP is used to indicate the deleterious SNPs in FLNB [48]. PolyPhen 1&2 (Polymorphism Phenotyping) tools measure an AA substitution’s influence on the protein function and structure [49]. Based on the fundamentals of the homology of the protein sequence and the physical properties of AA residues, SIFT (Sorting Intolerant from Tolerant) predicts the consequences of AA substitution on the protein function [50]. The functional impact of variants in the amino acid sequence was estimated by another computational tool, SNAP2 [51]. HOPE is a web-based tool used to determine the functional and structural impact of missense variants with a 3D structure. It resembles the systems mentioned earlier (PolyPhen, SIFT, ALAMUT) [52].

### 2.4. Stability Prediction

It is necessary to predict protein stability, as a missense variant results in the alteration of the protein structure and leads to loss of its function. The stability results of the variants were obtained using sequential input in the iStable tool [53]. I-Mutant 2.0 calculations can be made from either the protein sequence or the protein structure and are used for protein stability predictions of missense variants [48].

### 2.5. SNPeffect

SNPeffect is a server used for phenotyping analysis tools to anticipate the consequences of variants based on structural stability, protein aggregation, chaperone binding, and amyloid formation (FoldX, TANGO, LIMBO, and WALTZ) [54].

### 2.6. Preparation of Variant Models

The variants finalized for MDS were F161C, W148R, and L171R. To investigate the variants’ effect on the protein, the native protein (PDB ID: 2WA5) was mutated. Visualization and energy minimization of the structures was carried out using Swiss-PdbViewer, an integrated GROMOS96 force field [55,56].

### 2.7. Molecular Dynamics Simulation (MDS)

MDS for the native (PDB ID: 2WA5) and the three variants (W148R, F161C, and L171R) was performed using GROningen MAchine for Chemical Simulations (GROMACS); GROMOS96 54a7 force field [57,58]. The plugin option ‘ignh’ of GROMACS was used to ignore the hydrogen atoms [59]. The protein structure was set at a distance of 10 Å between the box and the protein threshold [60]. The sodium ions were used as counter ions for system neutralization [61,62,63]. The solvation of protein models was performed using Simple Point Charge water models in a cubic box [64]. With a calculated force of 1000.0 kJ/mol/nm, energy minimization was initiated for 5000 steps. Using the Particle Mesh Ewald approach, electrostatic corrections were performed [65,66], while bond length geometry was restricted using the system Steepest Descent Algorithm (SDA) [67]. The SETTLE algorithm was used to restrain the water molecule’s geometry [68], and the nonwater bonds were restricted using the LINCS algorithm [69]. After energy minimization, the NVT (temperature) and NPT (pressure) systems were fixed with 300 K using the Berendsen Coupling Method and performed for 500 ps [70]. Temperature and pressure normalization was conducted with the help of the Parrinello-Rahman barostat method [71]. The complete simulation of the system was conducted by 100 ns using the native and mutant models.

### 2.8. Trajectory Analysis

MDS was analyzed to explore the Root Mean Square Fluctuation (RMSF), Solvent Accessible Surface (SAS), Root Mean Square Variance (RMSD), Radius of Gyration (Rg) by utilizing gmx rms, gmx hbond, gmx gyrate, gmx sas, and gmx rmsf in GROMACS plugins. After completing the analysis, the graphs were plotted using Xmgrace software. The URLs are provided for the datasets and tool predictions (Table S5).

## 3. Results

### 3.1. Evolutionary Conservation Analysis

**Figure 1:**
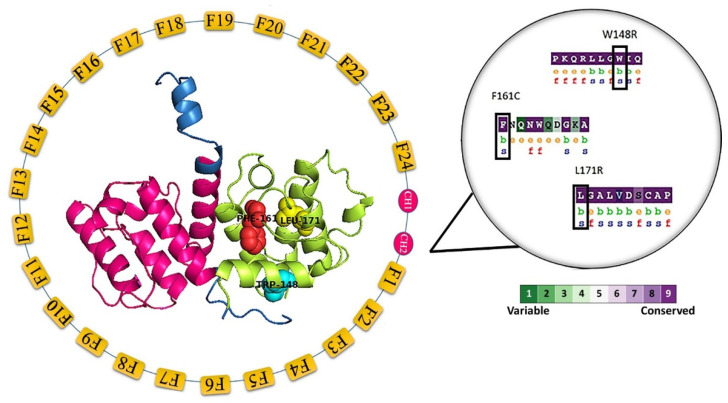
A schematic representation of a domain and the interpretation of the three variants’ evolutionary and conservation analysis (W148R, F161C, and L171R). The native protein structure (PDB ID: 2WA5) describes the variant sites in the center. The calponin-homology 1 (CH1; residues from 16–122) and calponin-homology 2 (CH2; residues from 139–242) are represented in magenta oval color; whereas repeated Filamin domains (F1–F24; residues from 249–2601) are represented in the yellow box. The variants W148R, F161C, and L171R are represented in cyan, red, and yellow spheres. The magnified image depicts all three variants that are structurally important and buried.

Evolutionary conservation analysis was carried out for all the 79 variants; six variants (W148R, F161C, L171Q, L171R, W165C, and G168C) that are present in the CH2 domain were shown to be functionally essential and showed a highly conserved score of 9 (Figure 1). Figure S1 depicts the conservation score for 2602 amino acids from FLNB protein.

### 3.2. Pathogenicity Prediction Analysis

A total of 79 *FLNB*-related variants were subjected to the pathogenicity study that comprised seven computational methods. SIFT predicted 71 variants of 79 variants as deleterious, Polyphen- 1 predicted 66 variants of 79 variants as deleterious, whereas PolyPhen-2 predicted 73 variants as deleterious. PredictSNP predicted 73 variants of 79 variants as deleterious. Out of 79 variants, MAPP predicted 37 variants as deleterious, PhD-SNP predicted 71 variants of 79 variants as deleterious, and SNAP predicted 53 variants of 79 variants as deleterious (Table S2).

### 3.3. Stability Prediction Analysis

Out of 79 variants, 53 showed decreased stability in iMutant 2.0 SEQ, and 64 showed decreased stability in MUpro and iStable, respectively (Table S3). Therefore, all 79 variants were subjected to biochemical and physicochemical characterization.

### 3.4. Biochemical and Physicochemical Characterization

Using the computational tool Align GVGD, all 79 variants were subjected to biochemical and physicochemical characterization. To conclude with the protein’s function, Align GVGD classified 59 variants as Class C65 (Table S4). All the variants were chosen for further evolutionary and conservation analysis after predicting the biochemical and physicochemical characterization method in silico. A consolidated table comparing all selected variants with tool predictions is provided in Table 1.

### 3.5. SNPeffect and HOPE

All six variants, which showed highly pathogenic and conserved scores, were subjected to SNPeffect analysis (Table 2). The TANGO results showed that none of the six variants (W148R, F161C, L171Q, L171R, W165C, and G168C) affected the protein’s aggregation tendency. Variant W148R alone decreased the protein’s amyloid propensity. The FoldX result shows that variants W165C, G168C, and L171R severely diminished the protein stability, while W148R, F161C, and L171Q lowered it to a lesser extent. LIMBO shows that none of the six variants had an impact on the protein’s chaperone binding phenomenon. The six variants in the CH2 domain were further subjected to HOPE server prediction. The HOPE server predicted that the variants reside in a critical domain for protein activity. The interaction disturbance between these domains due to these variants may affect protein function (Table 3).

### 3.6. Preparation of Protein Model

Among the six variants, only three (W148R, F161C, and L171R) showed severe phenotypes among patients and were selected for MDS. Before MDS was initiated, the native structure was mutated, and then energy was minimized with the usage of Swiss-PDBViewer.

### 3.7. Molecular Dynamics Simulation (MDS) and Trajectory Analysis

**Figure 2:**
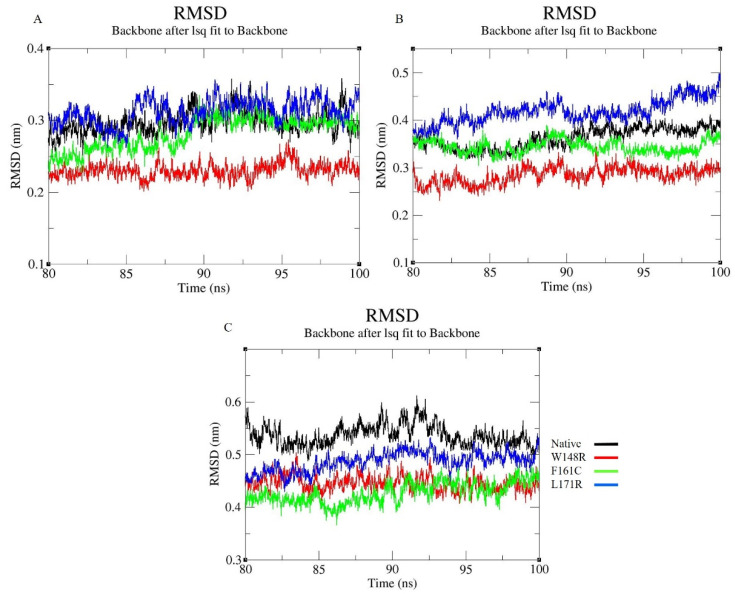
The block-based RMSD graph representing MDS for 80ns-100ns of native and mutants (**A**) CH1 domain (residues from 16–122); (**B**) CH2 domain (residues from 139–242); and (**C**) whole proteins, with X-axis representing time (ns) and Y-axis representing RMSD (nm). Color scheme: Native (Black), W148R (Red), F161C (Green), L171R (Blue).

To comprehend the effect of these variants on the protein’s function and structure, GROMACS 4.6.3 software was used to conduct MDS on the native and three shortlisted variants (W148R, F161C, and L171R). We analyzed the RMSD, RMSF, Rg, and SAS for the CH1 and CH2 domains. RMSF was plotted to analyze the native protein flexibility to compare it with the mutated protein’s flexibility. RMSD was plotted to examine the stability of the native protein to compare it with the stability of the mutated protein. The Rg and hydrogen bonds (H-bonds) were plotted to analyze the native and mutated protein’s compactness. The solvent-accessible surface was also used to compute the secondary structure of the protein. Protein stability was determined using RMSD parameters, and its value was calculated from the protein’s native structure. In a protein simulation, the structural stability of the protein is shown by deviation. The whole RMSD pattern was altered in the case of the mutant CH2 domain. Figure 2A and Figure S2A represent the RMSD graph of the CH1 domain, exhibiting deviated patterns in all the observed variants L171R (~0.2–0.35 nm) and F161C (~0.2–0.3 nm), whereas W148R showed convergence from 60ns. Figure 2B and Figure S2B present the RMSD graph of the CH2 domain, with more deviating patterns observed in all the three variants than the native. The block-based RMSD (Figure 2A,B) showed that all molecules had converged at the end of the simulation. By comparing the RMSD of the CH1 with CH2 domain for the native and mutant molecules, more deviating patterns were observed in F161C and L171R; also, the whole protein block-based RMSD yielded similar findings (Figure 2C). The mutated proteins W148R and F161C converged at the end of the simulation, whereas the mutated protein L171R has deviated from the other two mutated and the native protein (Figure S2C).

**Figure 3:**
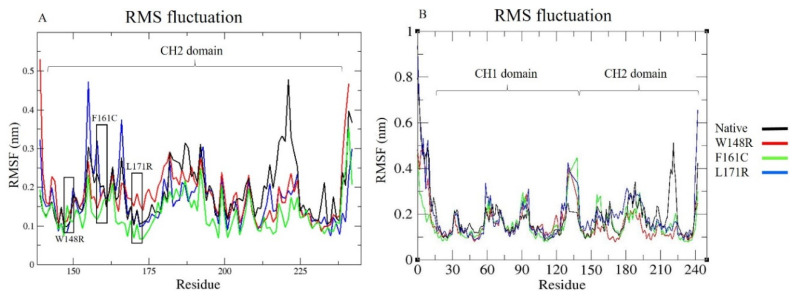
Root mean square fluctuation (RMSF) graph representing MDS for 100 ns of native and mutant (**A**) CH2 domain (residues from 139–242) and (**B**) whole proteins, with X-axis representing residues and Y-axis representing RMSF (nm). Color scheme: Native (Black), W148R (Red), F161C (Green), L171R (Blue). The black box represents the variant location.

The outcome of RMSD showed that the trajectories obtained a state of equilibrium and that they could be further used for the calculations of the subsequent analysis. Each protein’s nature is complex, and the structure’s flexibility is needed to sustain the protein dynamics. To estimate the flexibility of the mutated protein structure and a CH2 domain, the RMSF was determined. For the helix and sheets, the RMSF values were lower, whereas the RMSF values were higher for turns and bends. The CH2 domain mutants (W148R, F161C, and L171R) showed higher fluctuations in several regions than the native protein (Figure 3A). The overall RMSF was also computed for the native and mutant proteins. Between residues 40–55, 63–75, 210–240, the native protein showed higher fluctuations than all three variant proteins (Figure 3B).

**Figure 4:**
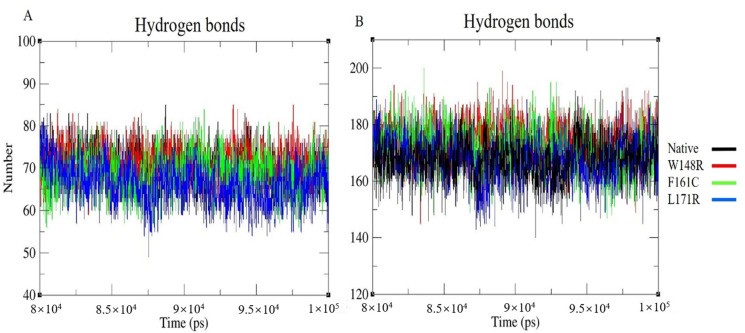
Graphical representation of the number of intramolecular H-bonds for last 20 ns of native and mutant (**A**) CH2 domain (residues from 139–242) and (**B**) whole proteins, with X-axis representing time (ps) and Y-axis was representing numbers. Color scheme: Native (Black), W148R (Red), F161C (Green), L171R (Blue).

**Figure 5:**
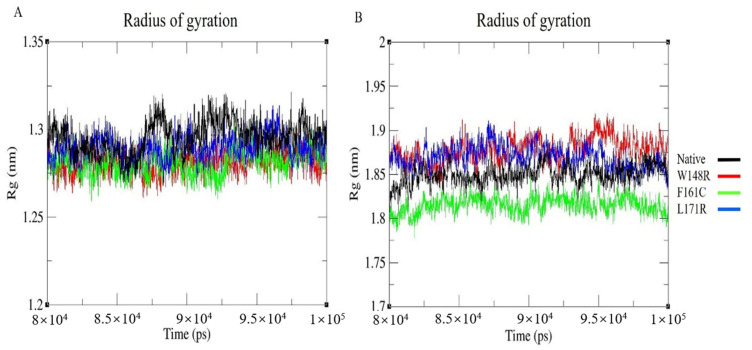
Radius of gyration (Rg) graph representing MDS for last 20 ns of native and mutant (**A**) CH2 domain (residues from 139–242) and (**B**) whole proteins, with X-axis representing time (ps) and Y-axis was representing Rg (nm). Color scheme: Native (Black), W148R (Red), F161C (Green), L171R (Blue).

It is necessary to understand the precise protein structure to evaluate the proper functioning of the protein; the RMSF result showed that the structural stability or the flexibility of the protein depends on the type of variant. Intramolecular H-bonds are critical in maintaining the protein’s stability. If more H-bonds exist, then the protein’s structural compactness will be higher. If the number of H-bonds is low, then the protein’s structural compactness will be lower. The intramolecular H-bonds were calculated to determine the conformational changes of the mutant protein/CH2-domain relative to the native protein/CH2-domain. As depicted in Figure 4A, the number of H-bonds in the mutant CH2 domain is more relative to the native CH2 domain, indicating that the compactness is higher in the mutant CH2 domain than the native CH2 domain. The block-based intramolecular H-bonds were plotted for whole proteins to identify the number of H-bonds formed at the end of the simulation. Here, ~165–175 H-bonds were formed in the native and L171R proteins, whereas the mutant models (W148R and F161C) formed more H-bonds comparison (Figure 4B). The intramolecular H-bonds were also plotted for the CH2 domain and whole protein and depicted in Figure S3A,B. The Rg was used to predict the compactness of the protein in the solution. The block-based Rg plot indicated that the mutants W148R and F161C in the CH2 domain showed higher fluctuation than mutant L171R in the CH2 domain and native CH2 domain (Figure 5A); this suggests that the mutant protein is more compact/rigid than the native protein and that this might lead to protein aggregation in patients with LS-AO-BD spectrum disorders. Figure 5B depicts the block-based Rg for the whole native and mutant proteins that exhibited overall higher deviation patterns for W148R and L171R compared to native and F161C models. The Rg was also computed for the CH2 domain and whole native and mutant proteins (W148R, F161C, and L171R). The results are depicted in Figure S4A,B.

### 3.8. Solvent Accessible Surface

**Figure 6:**
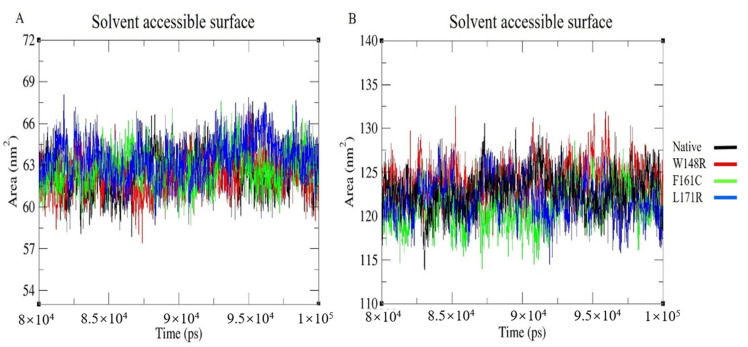
Solvent accessible surface (SAS) graph representing MDS for last 20 ns of native and mutant (**A**) CH2 domain (residues from 139–242) and (**B**) whole proteins, with X-axis representing time (ps) and Y-axis was representing Area (nm^2^). Color scheme: Native (Black), W148R (Red), F161C (Green), L171R (Blue).

The solvent-accessible surface computes the number of amino acids present in the protein’s surface and the hydrophobic core. This analysis further determined the amino acids that bind with neighboring solvent molecules. A change in the SAS might result in a change in the protein structure. All selected variants were located in the CH2 domain. The block-based SAS were plotted for the CH2 domain and whole protein models (Figure 6A,B). The W148R mutant of the CH2 domain exhibited higher fluctuation than the other two mutant CH2 domains (F161C and L171R) and the native-CH2 domain (Figure S5A). Similar results were obtained for the whole protein (Figure S5B). The change in surface amino acids might contribute to alterations in the mutant protein’s overall structure.

## 4. Discussion

Filamins (FLN) constitute a cytoplasmic family of three protein isoforms, FLNA, FLNB, and FLNC [14]. These proteins’ critical function is to assimilate cytoskeleton regulation and signaling phenomenon by establishing a network of F actin and other protein contacting molecules [20]. The FLN family protects the cellular structure’s stability by meshing the cytoskeleton F-actin into parallel bundles, providing support to the cell membrane. Filamins consist of an ABD in the N-terminal essential for actin binding and cell mechanoprotection, a C-terminal dimerization domain, and a central rod-like domain [72,73]. The primary variants in *FLNB* primarily occur in the domain (CH2) and around the hinge-1 region [19,20]. Four variants in FLNB that cause skeletal malformation disorders are autosomal dominant, often linked to missense or small in-frame deletions. The diseases are BD, AOI, AOIII, and LS [17].

To study the effect of each variant, we performed computational analysis. Additionally, we evaluated the consequences of different protein function variants. In this study, we collected a total of 79 variants from UniProt [38], HGMD [39], ClinVar [74] (Table S1). All 79 variants were subjected to pathogenicity prediction analysis. Predict SNP predicts the protein’s functionality from deleterious to neutral, and it comprises eight computational methods or tools. MAPP [45], PolyPhen1 [75], PolyPhen2 [49], SIFT [50], SNAP2 [76], PredictSNP [44] and PhD-SNP [48] servers predicted 37, 66, 73, 71, 53, 73, 71 variants as deleterious, respectively (Table S2). Variants were further subjected to the HOPE server [52], which identified six variants, W148R, F161C, L171Q, L171R, W165C, and G168C, located in a domain which is vital for the activity of the protein. Protein stability prediction is vital because a single amino acid change leads to modifications in the protein structure, contributing to a loss of function. In this study, all 79 variants were subjected to stability prediction. iStable tool [53], I-Mutant 2.0 [77] were used to compute the protein’s stability. Among the 79 variants, 53 showed decreased stability in iMutant 2.0 SEQ, and 64 showed decreased stability in MUpro and iStable, respectively (Table S3). All 79 were further subjected to biophysical and physicochemical characterization. Align GVGD [41] classified 59 variants as Class C65 (Table S4), and all variants were chosen for further evolutionary and conservation analysis using ConSurf [78]. The evolutionary conservation analysis was applied to all 79 variants; six variants (W148R, F161C, L171Q, L171R, W165C, and G168C) that are present in the CH2 domain were functionally important and showed a highly conserved score of 9. Some residues are not important for the protein’s structure and function, but some are functionally and structurally important for the protein [79,80]. In line with this phenomenon, among these six variants, three are structurally important, as predicted from the ConSurf (Figure 1 and Figure S1). The six variants that showed highly conserved scores were further subjected to SNPeffect analysis. The analysis showed that the variant W148R reduced the protein’s amyloid propensity, whereas the other five did not. The FoldX results showed that the variants W165C, G168C, and L171R severely lowered the protein’s stability. The results obtained from LIMBO showed that none of the six variants affected the protein’s chaperone binding phenomenon and that none of the six variants affected the protein’s aggregation tendency (TANGO) (Table 2). The six variants in the CH2 domain were further subjected to HOPE server prediction. The HOPE server predicted that the six variants play important protein domain activity and that interaction disturbance between these domains due to missense variant may affect protein function (Table 3).

Among the six variants that showed a highly conserved score, only those associated with severe phenotypes among patients (W148R, F161C, and L171R) were selected for MDS [35]. Before initiating MDS, the protein was mutated and then energy minimized with the help of Swiss-PdbViewer. They were subjected to MDS for 100 ns using the GROMACS 4.6.3 software. Larsen LJ et al. (1950) indicated that Larsen syndrome consolidates multiple joint displacements and specific craniofacial aberrations [81]. Notwithstanding the presence of the concentrated variant clusters which cause LS, the mechanism underlying this condition remains uncertain. Bicknell et al. (2007) reported that it is unclear if the variants interrupt protein interactions or promote novel Filamin B interactions [24]. The FLNB with a variant in ABD (W148R) can induce cellular F- actin modification, Septin 2, Myosin II, and focal adhesion [82]. W148R, along with the E227K variant, results in enhanced binding to the actin cytoskeleton. Hu et al. (2014) and Wilson et al. (2009) suggested that the irregular structure of the FLNB F-actin complex due to W148R may be attributed to dysfunctions in cells which are responsible for the development of the skeleton, such as chondrocytes and osteoblasts in which FLNB is expressed [83,84]. BD-associated variants S235P and K171R caused near-absolute localization within the actin of the variant proteins containing cytoplasmic focal accumulations in transient transfection experiments [19].

**Figure 7:**
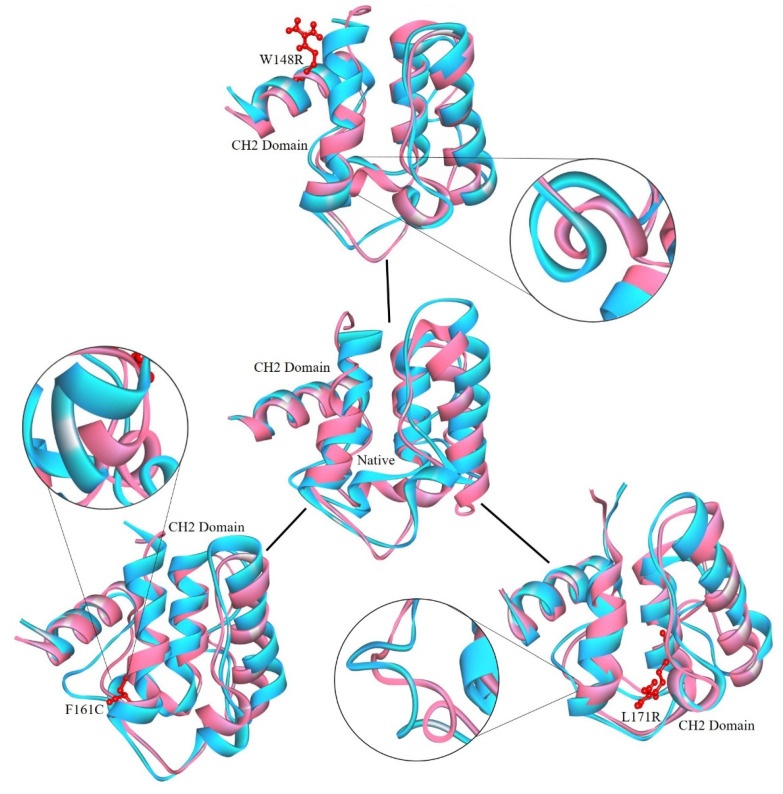
Conformational local shifts of the CH2 domain from the native and mutant FLNB protein’s MD trajectory. The secondary structure of 0ns (magenta) and the secondary structure of 100 ns (cyan) are depicted. The mutation sites are marked in red (ball and stick model), whereas the magnified circles represent the local changes in the CH2 domain due to variants.

The W148R and F161C mutants became converged at the end of the 100ns simulation, whereas the mutant protein L171R deviated from the other two mutant proteins and the native protein. The whole RMSD pattern was altered as a result of the variants (Figure 2A). In RMSF, the mutant proteins (W148R, F161C, and L171R) showed higher fluctuations than the native protein between the residues 120–150 (Figure 3A). The number of H-bonds in the mutant proteins (W148R, F161C, and L171R) was higher than in the native protein (Figure 4A), indicating more intramolecular H-bonds and might lead to a rigid structure of mutant proteins. We also observed a change in the compactness of the protein owing to these variants. Mutants W148R and L171R showed more considerable fluctuation than mutant F161C and the native protein (Figure 5B), suggesting that the W148R and L171R might affect the overall structure rather than merely inducing changes at the CH2 domain (Figure 5A). The SAS showed considerable fluctuation in the variant protein W148R compared to the other two mutants (F161C and L171R) and the native protein (Figure 6A). A block-based analysis of RMSD, RMSF, H-bonds, Rg, and SAS for the native and mutant proteins is depicted in Figure 2C, Figure 3B, Figure 4B, Figure 5B and Figure 6B. To further examine the local/global conformational shifts in the CH2 domain, we analyzed the MD trajectory (0 ns and 100 ns) of the native and mutant CH2 domain. From this analysis, we found a substantial local change in the mutant CH2 domain compared to the native CH2 domain (Figure 7). These results suggest that the variants positioned at 148, 161, and 171 alter the protein structural conformation. Also, we speculate that these pathogenic variants might dysregulate actin–filamin interactions as the predominant factor influencing these disease conditions [14,19,24,25].

Bicknell et al. (2007) suggested that patients affected by Larsen syndrome with the F161C mutant have midface hypoplasia, Scoliosis, Spatulate fingers, and accessory ossification center; most of these clinical phenotypes are also seen in other Larsen syndrome patients with different variants [24]. In FLNB ABD, two variants were predicted to lead to F161C and E227K amino acid substitutions [14]. At the C-terminus of the ABD and the flexibility between CH1 and CH2 loop, the main structural effects of W148R were seen because of the increase in overall temperature [20]. Zhao et al. (2015) suggested that the subfractionation assay revealed a more extensive aggregation of the FLNB ABD variants W148R and E227K on the cytoskeleton of the native protein [82]. Variants E227K and W148R cause cellular F-actin to be rearranged together with myosin II, focal adhesions, and septin 2. W148R-induced actin cytoskeleton restructuring is linked to delayed spatial migration of cells [82]. The clinical phenotype of AO1 includes the absence of fibulae, club foot, cleft palate, and respiratory distress [85,86]. FLNB L171R-expressing ATDC5 cells had globular aggregation of FLNB protein and increased cell apoptosis levels.

In contrast, *FLNB* G1586R-expressing ATDC5 cells had uniformly distributed FLNB protein and decreased cell migration; the clinical phenotypes include midface hypoplasia, vertebral hypoplasia, etc., absence of long bones [21,22]. Taken together, our analyses with the help of computational strategies revealed that the variants that occur at the CH2 domain might be severe, resulting in distinct phenotypes in LS-AO-BD spectrum disorders. Therefore, all three variants in the CH2 domain play a critical role in the structure and function of the FLNB protein and are the major causes of skeletal development disruption.

## 5. Conclusions

The FLN family maintains the integrity of the cellular structure and regulates signaling molecules. Despite several FLNB variants’ identification, their impact is not well understood in a structural and functional context. Studying the amino acid sequences of the resulting protein variants in the context of sequence and structure may help understand genotype-phenotype correlations. Experimental techniques take time to provide results. Thus, in this study, computational methods were adapted to obtain accurate predictions in a short time period. In silico tools were utilized to predict three variants (W148R, F161C, and L171R), prioritized according to experimental studies’ findings. The impact of these variants was also investigated using several computational strategies to assess protein stability and structure. Overall, the three variants W148R, F161C, and L171R were predicted to be responsible for the observed structural differences in the FLNB mutants compared to the native protein, resulting in a loss of stability and consequently leading to AOI, LS, and BD phenotypes. Our work suggests that computational analysis could help classify the genetic variants at the molecular level and assist in drug discovery.

## Figures and Tables

**Table 1:** Selected variants that exhibited deleterious and decreased from all prediction tools.

S.No	1	2	3	4	5	6
Variant	W148R	F161C	W165C	G168C	L171Q	L171R
PredictSNP	D1	D1	D1	D1	D1	D1
MAPP	D1	D1	D1	D1	D1	D1
PhD-SNP	D1	D1	D1	D1	D1	D1
PolyPhen-1	D1	D1	D1	D1	D1	D1
PolyPhen-2	D1	D1	D1	D1	D1	D1
SIFT	D1	D1	D1	D1	D1	D1
SNAP	D1	D1	D1	D1	D1	D1
i-Mutant2.0	D2	D2	D2	D2	D2	D2
MUpro	D2	D2	D2	D2	D2	D2
iStable	D2	D2	D2	D2	D2	D2

D1—Deleterious; D2—Decrease.

**Table 2:** Phenotypic effect prediction of FLNB variant (missense) using the SNPeffect server.

S.No	Variant	Tango	Waltz	Limbo	FoldX
1	W148R	T.1	W.2	L.1	Reduces the protein stability.
2	F161C	T.1	W.1.	L.1	Reduces the protein stability.
3	W165C	T.1	W.1.	L.1	Severely reduces the protein stability.
4	G168C	T.1	W.1.	L.1	Severely reduces the protein stability.
5	L171Q	T.1	W.1.	L.1	Reduces the protein stability.
6	L171R	T.1	W.1.	L.1	Severely reduces the protein stability.

T.1 = Does not affect the aggregation tendency of your protein; W.1 = Does not affect the amyloid propensity of your protein; W.2 = Decreases the amyloid propensity of your protein; L.1 = Does not affect the chaperone binding tendency of your protein.

**Table 3:** Structural and functional impact of FLNB variant using the HOPE server.

S.No	Variant	Change in AA Property	Mutation Impact
1	W148R	The mutation introduces an amino acid with different properties	The mutant residue is smaller than the wild-type residue. The mutation will cause a possible loss of external interactions
2	F161C	The mutation introduces an amino acid with different properties	The mutant residue is smaller than the wild-type residue. The mutation will cause a possible loss of external interactions
3	W165C	The mutation introduces an amino acid with different properties	The mutant residue is smaller than the wild-type residue. The mutation will cause a possible loss of external interactions
4	G168C	Mutation of this glycine can abolish the protein function.	The wild-type residue was buried in the core of the protein. The mutant residue is bigger and probably will not fit.
5	L171Q	The mutation introduces an amino acid with different properties	The wild-type residue was buried in the core of the protein. The mutant residue is bigger and probably will not fit.
6	L171R	The mutation introduces an amino acid with different properties	The wild-type residue was buried in the core of the protein. The mutant residue is bigger and probably will not fit.

## Supplementary Materials

The following are available online, Figure S1: Evolutionary conservation analysis for FLNB protein, Figure S2: Root mean square deviation (RMSD) graph representing MDS for 100 ns of native and mutants, Figure S3: Graphical representation of the number of intramolecular H-bonds for 100 ns of native and mutants, Figure S4: Radius of gyration (Rg) graph representing MDS for 100 ns of native and mutants, Figure S5: Solvent accessible surface (SAS) graph representing MDS for 100 ns of native and mutants, Table S1: List of FLNB variants retrieved from UniProt and HGMD, Table S2: Pathogenicity analysis result of FLNB missense variants using PredictSNP server, Table S3: Stability prediction result of FLNB missense variants using iStable server, Table S4: Biochemical and physicochemical characterization of FLNB variants (missense) using Align GVGD server, Table S5: The URLs are provided for the datasets and tool predictions.

## Abbreviations

LS—Larsen syndrome; BD—Boomerang dysplasia; AO—Atelosteogenesis type I; ABD—Actin-Binding Domain; SCT—Spondylocarpotarsal Synostosis; HGMD—Human Gene Mutation Database; HOPE—Have (y)Our Protein Explained; GROMACS—GROningen MAchine for Chemical Simulations; SAS—Solvent Accessible Surface; MDS—Molecular Dynamics Simulation; FLNB—Filamin B; RMSD—Root Mean Square Deviation; RMSF—Root Mean Square Fluctuation; Rg—Radius of Gyration; CH1/CH2—Calponin-Homology.
