# Supplementary material for: Deciphering the Role of Filamin B Calponin-Homology Domain in Causing the Larsen Syndrome, Boomerang Dysplasia, and Atelosteogenesis Type I Spectrum Disorders via a Computational Approach
Source: Molecules. 2020 Nov 26;25(23):5543. doi: 10.3390/molecules25235543 (PMC7730838; doi:10.3390/molecules25235543)
Supplement: Supplementary file 1 [file molecules-25-05543-s001.zip › FLNB_Supplementary_Figure_988371.docx]

**Manuscript ID-988371**

**Title:** Deciphering the role of filamin B calponin-homology domain in causing the Larsen syndrome, Boomerang dysplasia, and Atelosteogenesis type I spectrum disorders via computational approach

**Supplementary Figures**


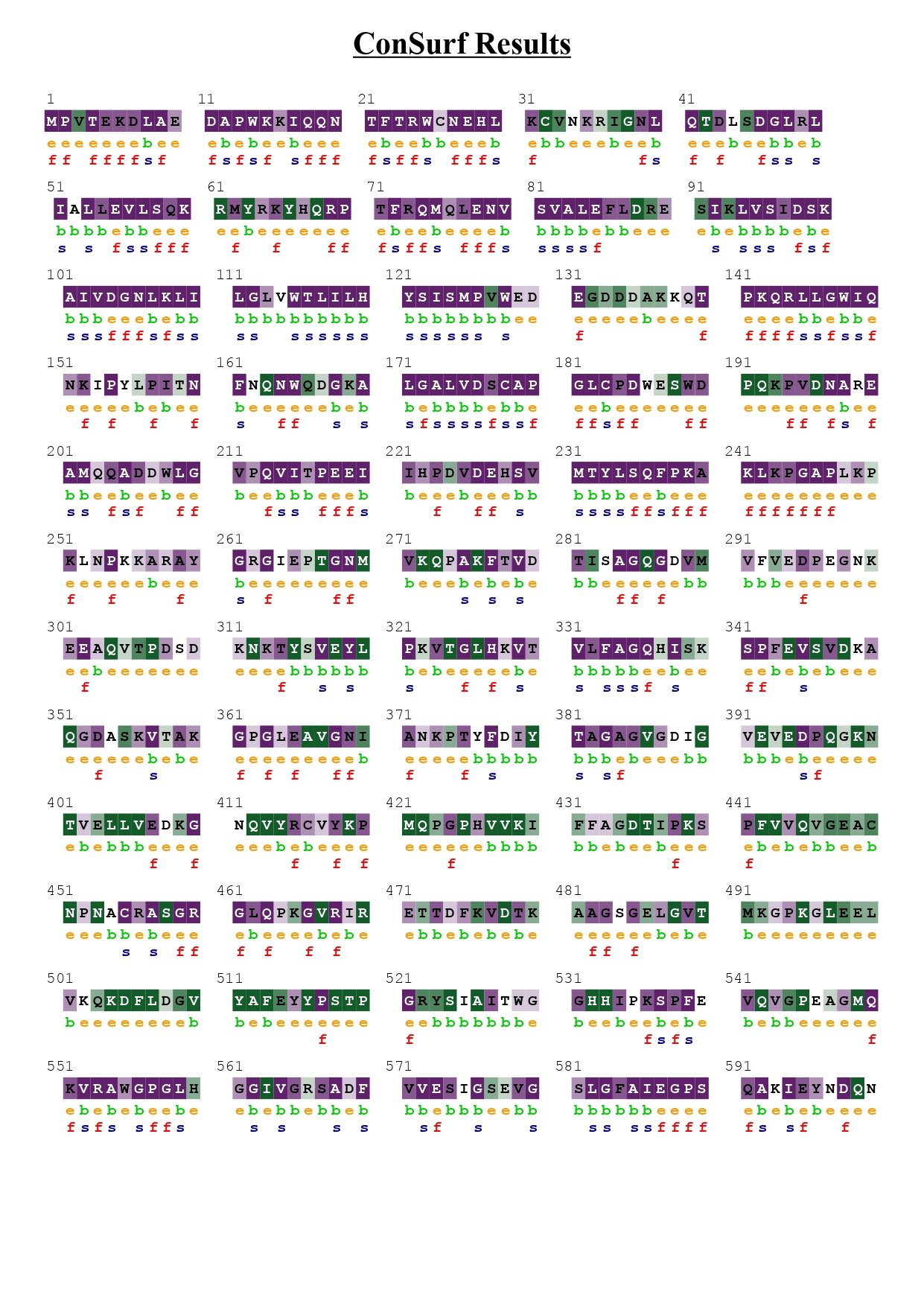


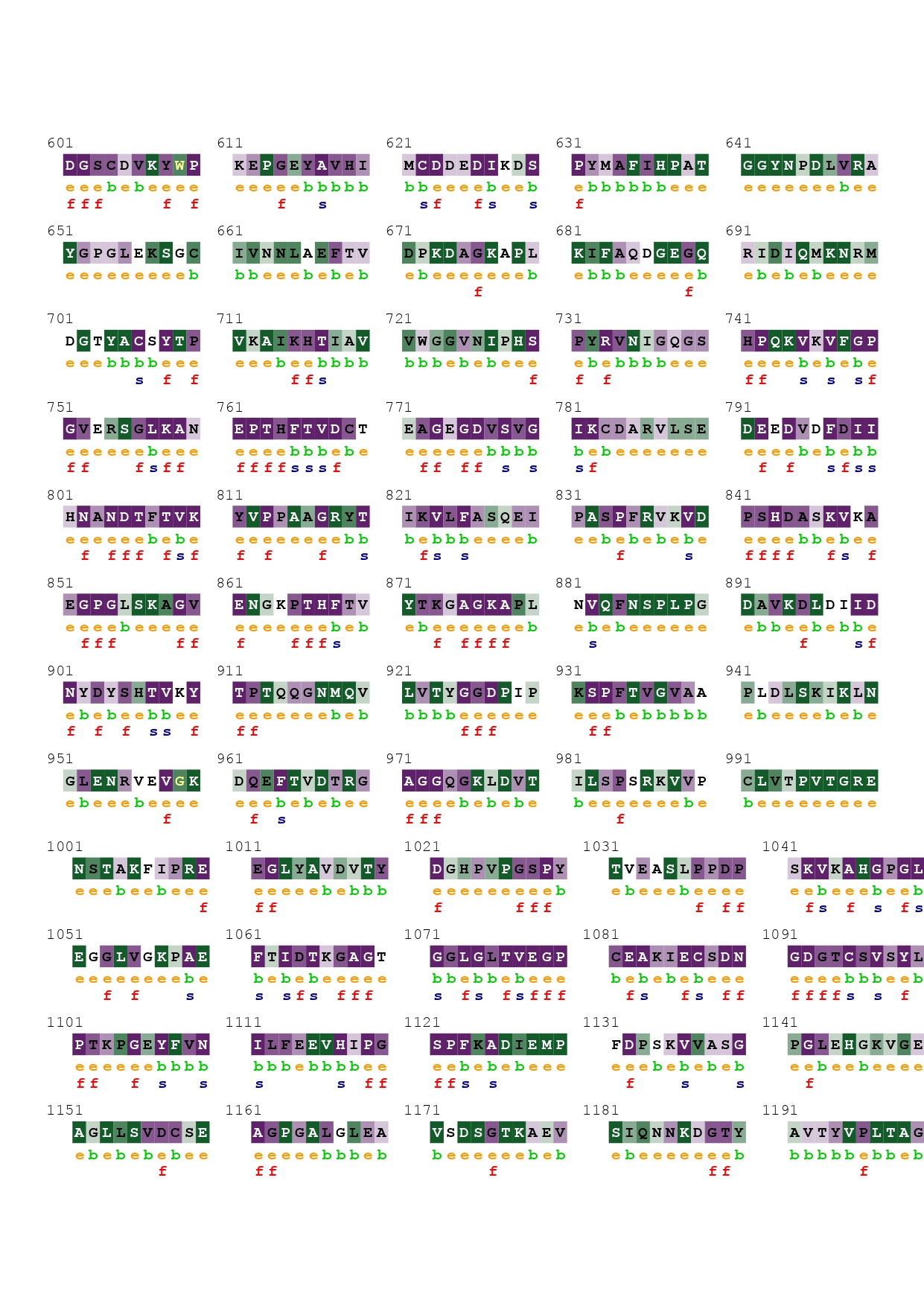


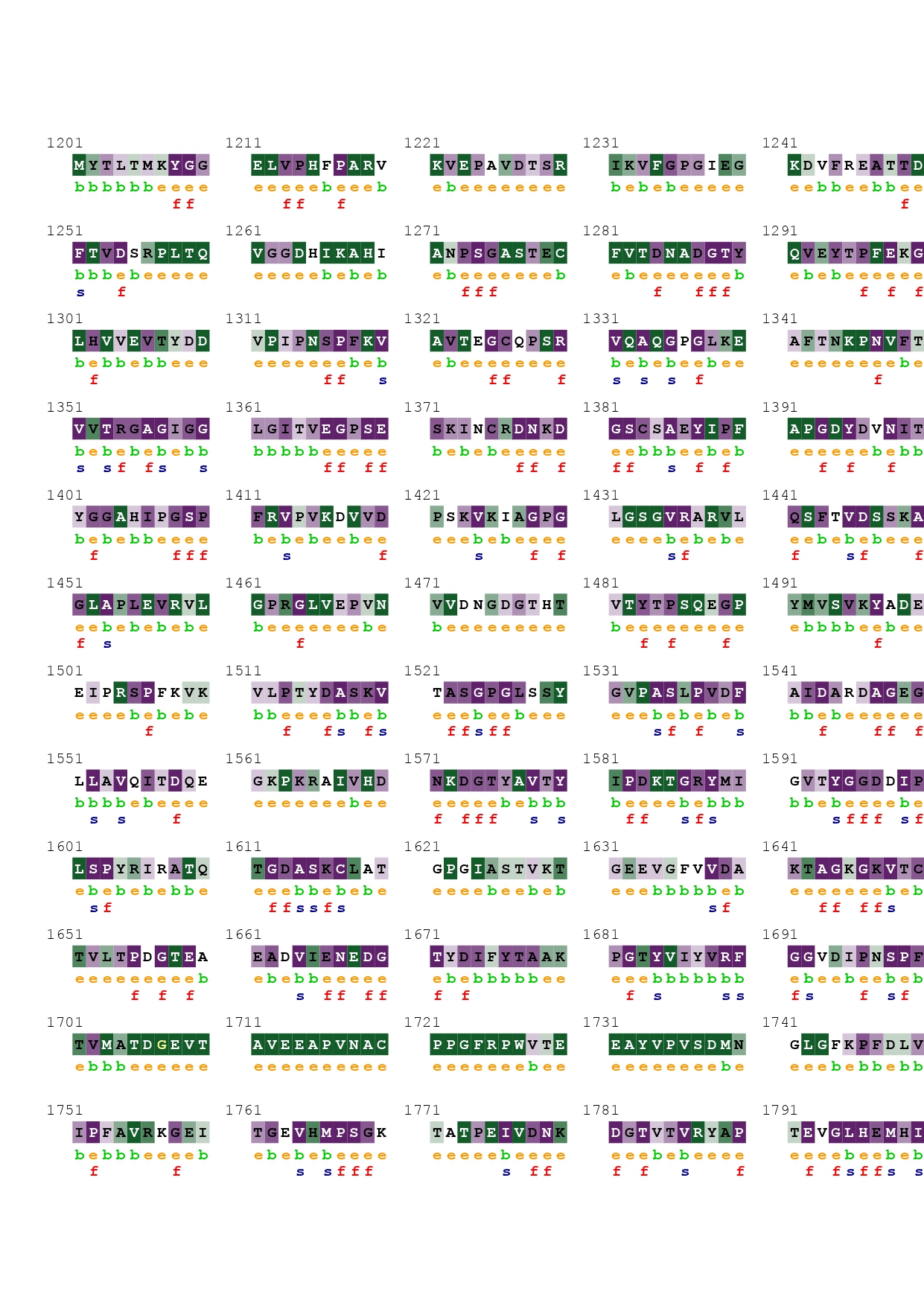


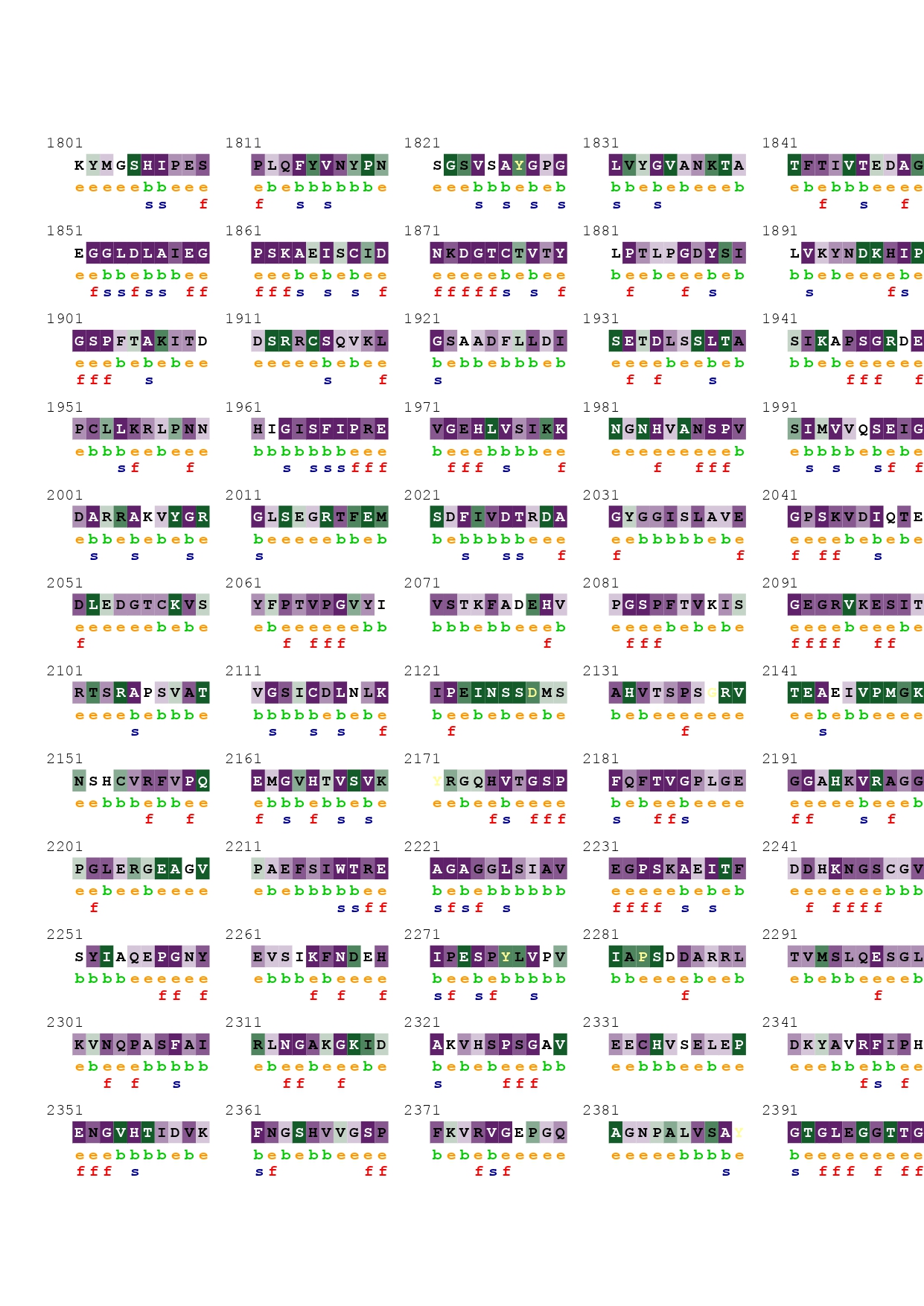


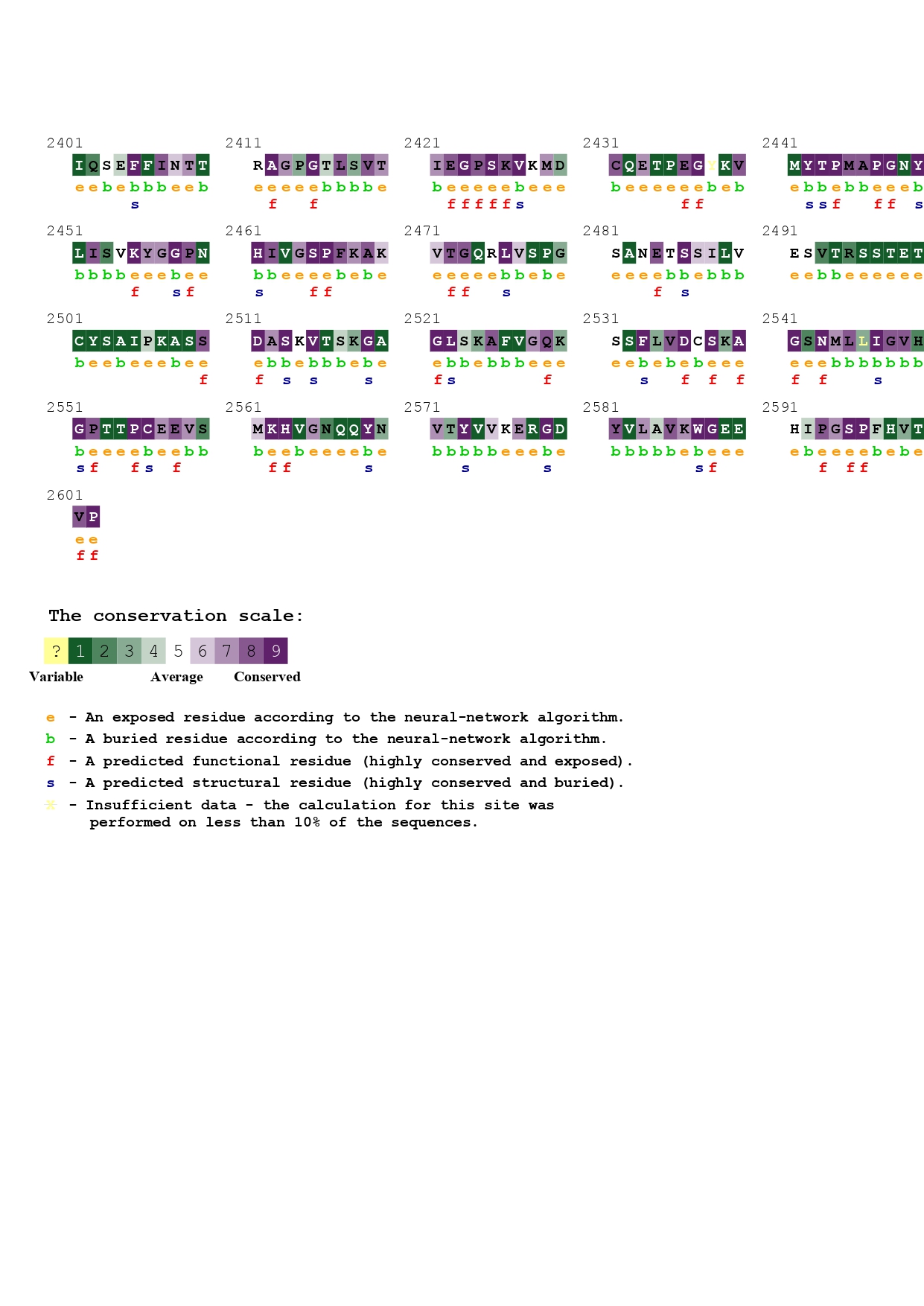


**Figure S1.** Evolutionary conservation analysis for FLNB protein. The conservation score for 2,602 amino acids for FLNB protein using ConSurf web-server. The conservation scale (variable/average/conserved) and important features of the amino acids (e/b/f/s) were provided.


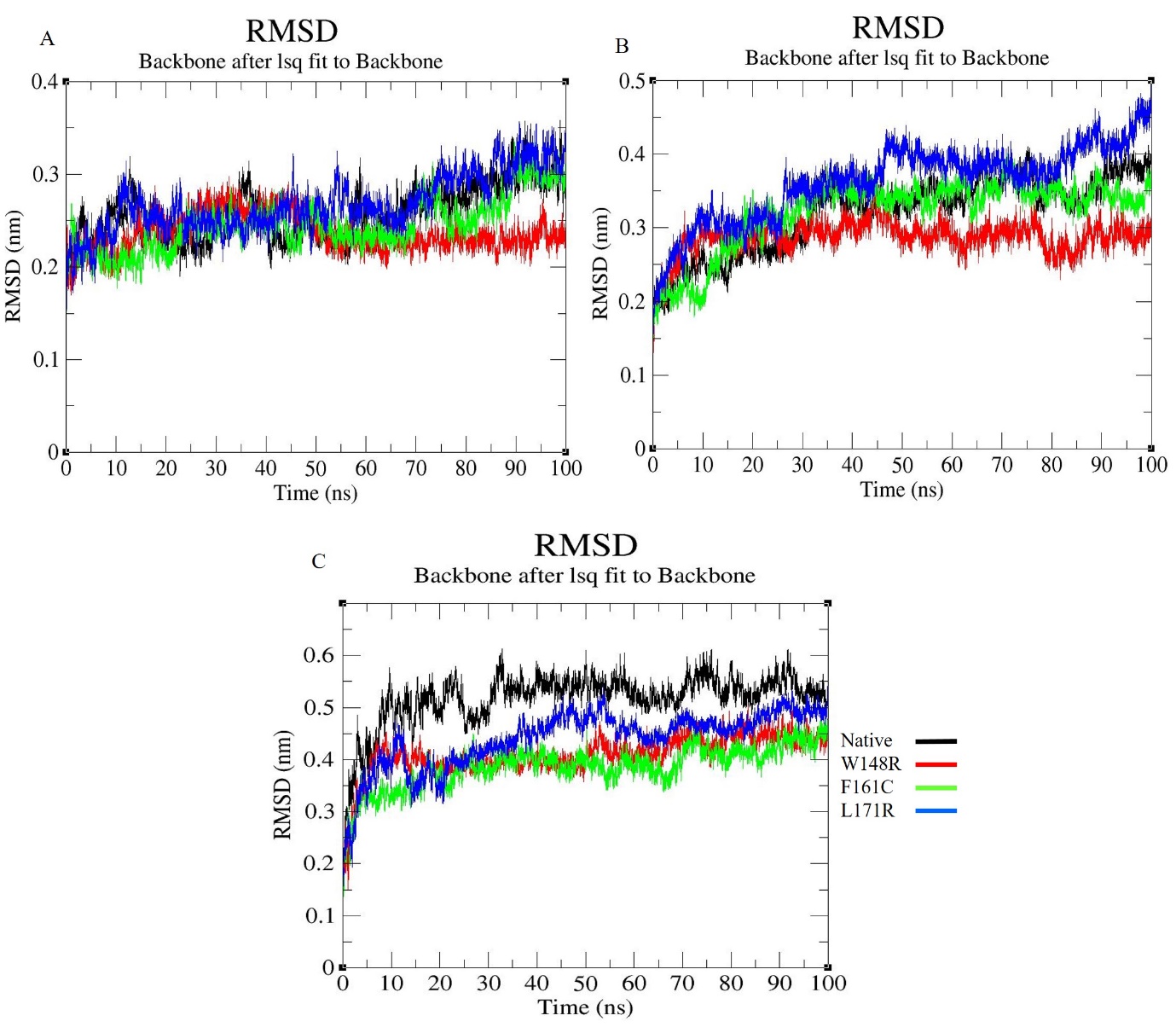


**Figure S2.** Root mean square deviation (RMSD) graph representing MDS for 100ns of native and mutant (A) CH1 domain (residues from 16-122); (B) CH2 domain (residues from 139-242); and (C) whole proteins, with X-axis representing time (ns) and Y-axis representing RMSD (nm). Color scheme: Native (Black), W148R (Red), F161C (Green), L171R (Blue).


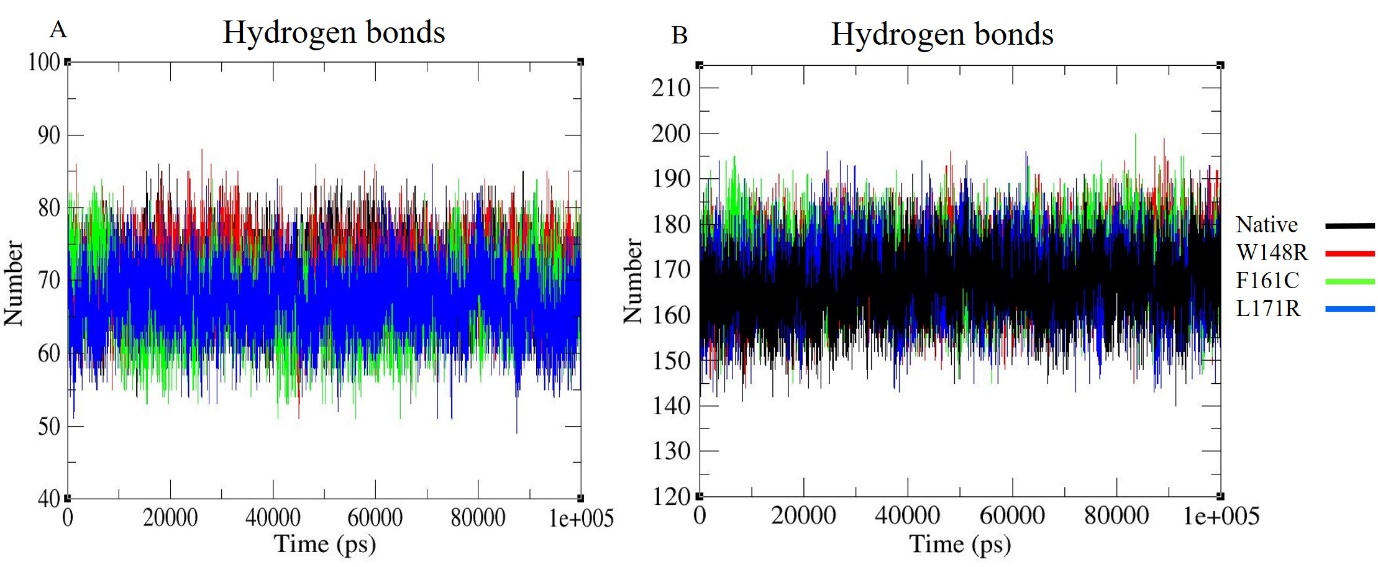


**Figure S3.** Graphical representation of the number of intramolecular H-bonds for 100ns of native and mutant (A) CH2 domain (residues from 139-242) and (B) whole proteins, with X-axis representing time (ps) and Y-axis representing Numbers. Color scheme: Native (Black), W148R (Red), F161C (Green), L171R (Blue).


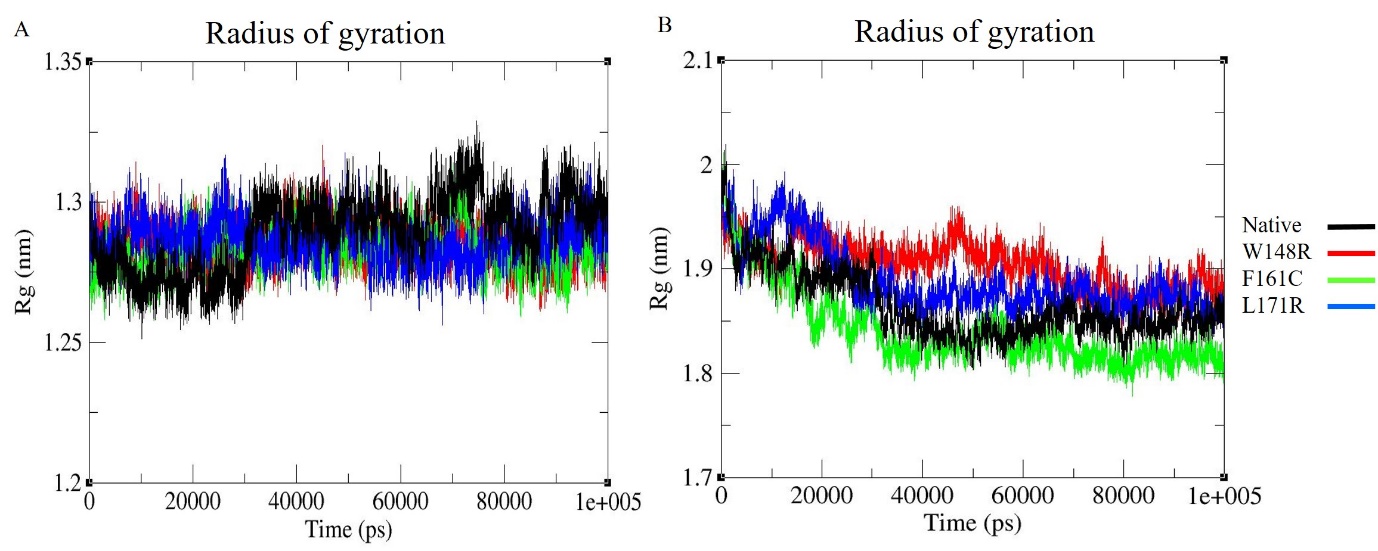


**Figure S4.** Radius of gyration (Rg) graph representing MDS for 100ns of native and mutant (A) CH2 domain (residues from 139-242) and (B) whole proteins, with X-axis representing time (ps) and Y-axis representing Rg (nm). Color scheme: Native (Black), W148R (Red), F161C (Green), L171R (Blue).


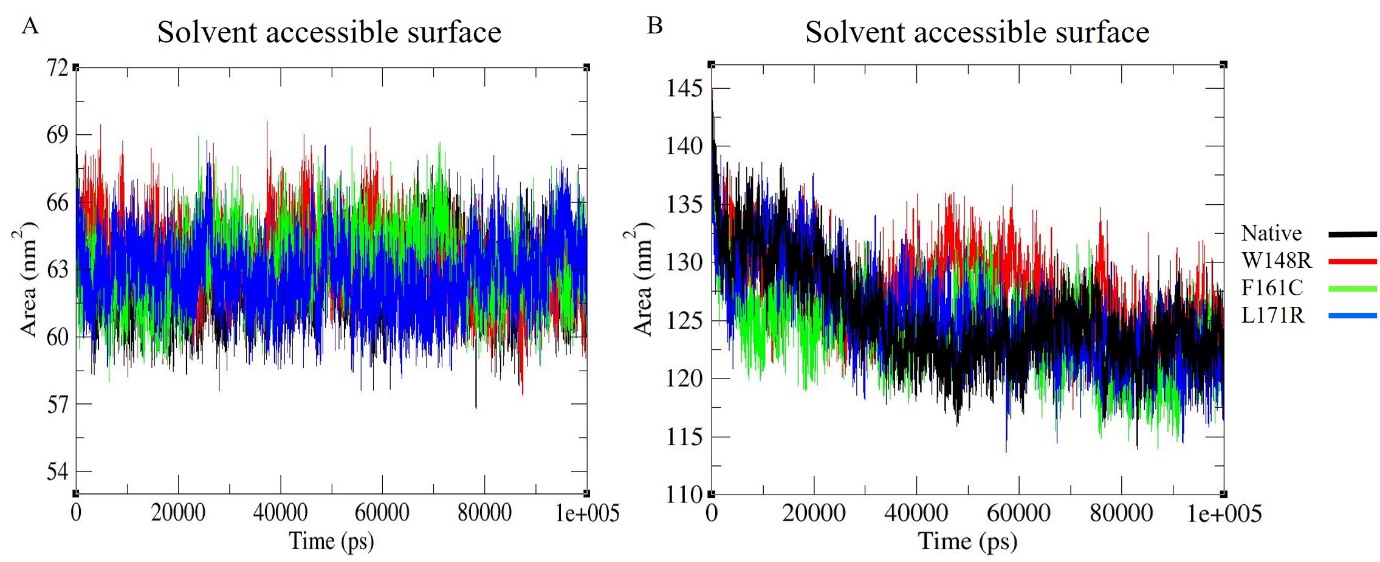


**Figure S5.** Solvent accessible surface (SAS) graph representing MDS for 100ns of native and mutant (A) CH2 domain (residues from 139-242) and (B) whole proteins, with X-axis representing time (ps) and Y-axis representing Area (nm2). Color scheme: Native (Black), W148R (Red), F161C (Green), L171R (Blue).
