# Supplementary material for: Deciphering the Role of Filamin B Calponin-Homology Domain in Causing the Larsen Syndrome, Boomerang Dysplasia, and Atelosteogenesis Type I Spectrum Disorders via a Computational Approach
Source: Molecules. 2020 Nov 26;25(23):5543. doi: 10.3390/molecules25235543 (PMC7730838; doi:10.3390/molecules25235543)
Supplement: Supplementary file 1 [file molecules-25-05543-s001.zip › FLNB_Supplementary_Table_988371.docx]

**Manuscript ID-988371**

**Title:** Deciphering the role of filamin B calponin-homology domain in causing the Larsen syndrome, Boomerang dysplasia, and Atelosteogenesis type I spectrum disorders via computational approach

**Table S1. List of FLNB variants retrieved from UniProt and HGMD.**

| **S.No** | **Accession ID** | **Variants** |
| --- | --- | --- |
| **1.** | **CM062740** | **W148R** |
| 2. | CM061770 | N160T |
| **3.** | **CM040994/VAR_033069** | **F161C** |
| 4. | CM122607 | Q163P |
| **5.** | **CM122597** | **W165C** |
| 6. | CM122608 | D167E |
| 7. | CM122609 | D167G |
| 8. | CM122603 | D167V |
| 9. | CM062734/VAR_033070 | G168S |
| 10. | CM122598 | G168V |
| **11.** | **CM122604** | **G168C** |
| 12. | CM085436 | A170P |
| **13.** | **CM062735** | **L171Q** |
| **14.** | **CM052234/VAR_033071** | **L171R** |
| 15. | CM143666 | A173T |
| 16. | CM040995/VAR_033072 | A173V |
| 17. | CM131740 | G181R |
| 18. | CM131739 | G181D |
| 19. | CM062738 | G181V |
| 20. | CM062742 | C183W |
| 21. | CM040996/VAR_033073 | S188P |
| 22. | CM122600 | W189R |
| 23. | CM122599 | W189G |
| 24. | CM122610 | P191L |
| 25. | CM122611 | N197I |
| 26. | CM062736 | A201V |
| 27. | CM122596 | M202T |
| 28. | CM040997/VAR_033074 | M202V |
| 29. | CM062733 | Q203P |
| 30. | CM122601 | A205T |
| 31. | CM122612 | W208R |
| 32. | CM062739 | G210V |
| 33. | CM122613 | I221F |
| 34. | CM040998/VAR_033075 | E227K |
| 35. | CM122614 | S229P |
| 36. | CM070930/VAR_033076 | L234V |
| 37. | CM052235/VAR_033077 | S235P |
| 38. | CM070928/VAR_033078 | G361S |
| 39. | CM122616 | G361D |
| 40. | CM122615 | G361C |
| 41. | CM122605 | G363R |
| 42. | CM070932/VAR_033079 | G363E |
| 43. | VAR_035917 | R566Q |
| 44. | VAR_035918 | N663K |
| 45. | VAR_035919 | T703K |
| 46. | CM041000/VAR_033080 | G751R |
| 47. | VAR_017182 | V1018M |
| 48. | VAR_017183 | D1157N |
| 49. | VAR_031392 | E1179K |
| 50. | CM1314442 | R1219W |
| 51. | VAR_033081/CM070931 | L1431R |
| 52. | VAR_031393 | V1471M |
| 53. | CM122617 | L1527H |
| 54. | CM122618 | L1527P |
| 55. | VAR_035920 | A1534G |
| 56. | CM061771 | S1535N |
| 57. | CM122619 | A1541P |
| 58. | CM122620 | I1542T |
| 59. | CM061773 | A1547D |
| 60. | CM041002/VAR_033083 | G1586R |
| 67. | VAR_033084/CM070933 | V1592D |
| 62. | CM122621 | Y1594S |
| 63. | CM122622 | I1599F |
| 64. | CM062743 | S1602P |
| 65. | CM122623 | S1602Y |
| 66. | VAR_033085/CM070929 | P1603L |
| 67. | CM062737 | G1612D |
| 68. | CM122717 | A1643P |
| 69. | CM062741 | A1643S |
| 70. | CM061772 | G1644W |
| 71. | CM122624 | G1646S |
| 72. | CM041004/VAR_033086 | G1691S |
| 73. | CM122626 | G1691D |
| 74. | CM122625 | G1691C |
| 75. | CM122606 | G1692S |
| 76. | CM122602 | P1699S |
| 77. | CM070934/VAR_033087 | G1834R |
| 78. | CM1314443 | D1870G |
| 79. | CM122627 | S1902R |

Variants mentioned in bold are predicted to be highly conserved from ConSurf analysis

**Table S2. Pathogenicity analysis result of FLNB missense variants using PredictSNP server.**

| **S.No** | **Variants** | **PredictSNP** | **MAPP** | **PhD-SNP** | **PolyPhen-1** | **PolyPhen-2** | **SIFT** | **SNAP** |
| --- | --- | --- | --- | --- | --- | --- | --- | --- |
| **1.** | **W148R** | **D** | **D** | **D** | **D** | **D** | **D** | **D** |
| 2. | N160T | D | D | D | D | D | D | N |
| **3.** | **F161C** | **D** | **D** | **D** | **D** | **D** | **D** | **D** |
| 4. | Q163P | D | D | D | N | D | D | N |
| **5.** | **W165C** | **D** | **D** | **D** | **D** | **D** | **D** | **D** |
| 6. | D167E | D | D | D | D | D | D | N |
| 7. | D167G | D | D | D | D | D | D | N |
| 8. | D167V | D | D | D | D | D | D | N |
| 9. | G168S | D | D | D | D | D | D | N |
| 10. | G168V | D | D | D | D | D | D | N |
| **11.** | **G168C** | **D** | **D** | **D** | **D** | **D** | **D** | **D** |
| 12. | A170P | D | N | D | D | D | D | D |
| **13.** | **L171Q** | **D** | **D** | **D** | **D** | **D** | **D** | **D** |
| **14.** | **L171R** | **D** | **D** | **D** | **D** | **D** | **D** | **D** |
| 15. | A173T | D | D | D | D | D | D | N |
| 16. | A173V | D | D | D | D | D | D | D |
| 17. | G181R | D | N | D | D | D | D | D |
| 18. | G181D | D | N | N | D | D | D | D |
| 19. | G181V | D | D | D | D | D | D | N |
| 20. | C183W | D | D | D | D | D | D | D |
| 21. | S188P | D | D | D | D | D | N | N |
| 22. | W189R | D | D | D | D | D | D | D |
| 23. | W189G | D | D | D | D | D | D | D |
| 24. | P191L | D | N | N | D | D | D | N |
| 25. | N197I | D | N | D | D | D | D | N |
| 26. | A201V | D | D | D | D | D | D | N |
| 27. | M202T | D | D | D | D | D | D | N |
| 28. | M202V | D | D | D | D | D | D | N |
| 29. | Q203P | D | D | D | D | D | D | D |
| 30. | A205T | D | D | D | D | D | D | N |
| 31. | W208R | D | N | D | N | D | D | D |
| 32. | G210V | D | D | D | D | D | D | N |
| 33. | I221F | N | N | N | N | D | D | N |
| 34. | E227K | D | D | D | D | D | D | N |
| 35. | S229P | D | D | D | D | D | D | N |
| 36. | L234V | N | D | N | N | D | N | N |
| 37. | S235P | D | D | D | D | D | D | D |
| 38. | G361S | D | N | D | D | D | D | D |
| 39. | G361D | D | N | D | D | D | D | D |
| 40. | G361C | D | N | D | D | D | D | D |
| 41. | G363R | D | D | D | D | D | D | D |
| 42. | G363E | D | N | D | D | D | D | D |
| 43. | R566Q | N | N | N | N | N | N | N |
| 44. | N663K | N | N | N | N | N | D | N |
| 45. | T703K | D | N | D | D | D | D | D |
| 46. | G751R | D | D | D | D | D | D | D |
| 47. | V1018M | D | U | D | D | D | D | D |
| 48. | D1157N | D | U | D | N | D | N | D |
| 49. | E1179K | N | U | N | N | N | N | D |
| 50. | R1219W | D | U | D | D | D | D | D |
| 51. | L1431R | D | U | D | D | D | D | D |
| 52. | V1471M | D | U | D | D | D | N | D |
| 53. | L1527H | D | U | D | D | D | D | D |
| 54. | L1527P | D | U | D | D | D | D | D |
| 55. | A1534G | N | U | D | N | N | D | N |
| 56. | S1535N | N | U | N | N | N | D | D |
| 57. | A1541P | D | D | D | N | N | D | D |
| 58. | I1542T | D | D | D | D | D | N | N |
| 59. | A1547D | D | D | D | D | D | D | D |
| 60. | G1586R | D | U | D | D | D | D | D |
| 67. | V1592D | D | U | D | D | D | D | D |
| 62. | Y1594S | D | U | D | D | D | D | N |
| 63. | I1599F | D | U | D | D | D | D | D |
| 64. | S1602P | D | U | D | D | D | D | D |
| 65. | S1602Y | D | U | D | D | D | D | D |
| 66. | P1603L | D | U | D | D | D | D | D |
| 67. | G1612D | D | U | D | D | D | D | D |
| 68. | A1643P | D | U | D | D | D | D | D |
| 69. | A1643S | D | U | D | D | D | D | D |
| 70. | G1644W | D | U | D | D | D | D | D |
| 71. | G1646S | D | N | N | D | D | D | D |
| 72. | G1691S | D | N | D | N | D | D | D |
| 73. | G1691D | D | N | D | N | D | D | D |
| 74.. | G1691C | D | N | D | D | D | D | D |
| 75. | G1692S | D | N | D | D | D | D | D |
| 76. | P1699S | D | U | D | D | D | N | D |
| 77. | G1834R | D | D | D | D | D | D | D |
| 78. | D1870G | D | N | D | D | D | D | D |
| 79. | S1902R | D | D | D | D | D | D | D |

D-deleterious; N-Neutral; U-Unknown; Variants mentioned in bold are predicted to be highly conserved from ConSurf analysis

**Table S3. Stability prediction result of FLNB missense variants using iStable server.**

| **S.No** | **Variants** | **i-Mutant2.0 SEQ** | **DDG** | **MUpro** | **Conf. score** | **iStable** | **Conf. score** |
| --- | --- | --- | --- | --- | --- | --- | --- |
| **1** | **W148R** | **D** | **-0.49** | **D** | **-0.54** | **D** | **0.79** |
| 2 | N160T | I | 0.64 | D | -0.05 | D | 0.51 |
| **3** | **F161C** | **D** | **-0.92** | **D** | **-0.63** | **D** | **0.73** |
| 4 | Q163P | D | -0.47 | D | -0.08 | D | 0.74 |
| **5** | **W165C** | **D** | **-1.05** | **D** | **-0.26** | **D** | **0.87** |
| 6 | D167E | I | 0.32 | D | -0.66 | D | 0.61 |
| 7 | D167G | I | -0.15 | D | -1.00 | D | 0.68 |
| 8 | D167V | D | 0.32 | D | -0.58 | D | 0.84 |
| 9 | G168S | D | -1.1 | D | -1.00 | D | 0.79 |
| 10 | G168V | N | -0.43 | D | -0.46 | D | 0.72 |
| **11** | **G168C** | **D** | **-0.75** | **D** | **-0.63** | **D** | **0.78** |
| 12 | A170P | D | -0.11 | D | -0.16 | D | 0.82 |
| **13** | **L171Q** | **D** | **-1.29** | **D** | **-1.00** | **D** | **0.86** |
| **14** | **L171R** | **D** | **-1.15** | **D** | **-1.00** | **D** | **0.85** |
| 15 | A173T | D | -0.16 | D | -1.00 | D | 0.76 |
| 16 | A173V | I | 0.43 | D | -0.30 | I | 0.55 |
| 17 | G181R | D | -0.1 | I | 0.39 | D | 0.62 |
| 18 | G181D | D | -0.79 | I | 0.71 | D | 0.62 |
| 19 | G181V | D | -0.41 | I | 0.11 | D | 0.67 |
| 20 | C183W | I | 0.35 | D | -0.26 | I | 0.50 |
| 21 | S188P | I | 0.18 | D | -0.77 | D | 0.63 |
| 22 | W189R | D | N | D | -1.00 | D | 0.74 |
| 23 | W189G | D | -1.45 | D | -1.00 | D | 0.80 |
| 24 | P191L | I | 0.2 | D | -0.53 | D | 0.53 |
| 25 | N197I | I | 1.83 | D | -0.24 | D | 0.56 |
| 26 | A201V | D | -0.34 | D | -0.05 | D | 0.73 |
| 27 | M202T | D | -0.16 | D | -1.00 | D | 0.78 |
| 28 | M202V | D | -0.69 | D | -1.00 | D | 0.78 |
| 29 | Q203P | D | -0.47 | D | -0.16 | D | 0.84 |
| 30 | A205T | D | -0.45 | D | -0.55 | D | 0.89 |
| 31 | W208R | D | -0.63 | D | -1.00 | D | 0.69 |
| 32 | G210V | D | -0.27 | D | -0.51 | D | 0.77 |
| 33 | I221F | D | -0.69 | D | -1.00 | D | 0.90 |
| 34 | E227K | D | -0.09 | D | -1.00 | D | 0.75 |
| 35 | S229P | I | -0.04 | I | 0.67 | I | 0.65 |
| 36 | L234V | D | -1.39 | D | -0.82 | D | 0.80 |
| 37 | S235P | I | 0.12 | D | -0.27 | D | 0.52 |
| 38 | G361S | N | -1.19 | D | -0.65 | D | 0.71 |
| 39 | G361D | D | -0.98 | D | -0.07 | D | 0.77 |
| 40 | G361C | D | -1.01 | D | -0.84 | D | 0.79 |
| 41 | G363R | D | -0.58 | I | 1.00 | D | 0.52 |
| 42 | G363E | I | -0.77 | I | 1.00 | I | 0.71 |
| 43 | R566Q | D | -0.26 | D | -0.92 | D | 0.59 |
| 44 | N663K | D | -0.39 | I | 0.36 | I | 0.71 |
| 45 | T703K | D | -0.69 | D | -1.00 | D | 0.74 |
| 46 | G751R | I | -0.27 | I | 1.00 | I | 0.78 |
| 47 | V1018M | D | -1.35 | D | -0.12 | D | 0.69 |
| 48 | D1157N | D | -0.58 | D | -1.00 | D | 0.74 |
| 49 | E1179K | N | -0.48 | D | -1.00 | D | 0.76 |
| 50 | R1219W | I | 0.43 | D | -0.95 | D | 0.53 |
| 51 | L1431R | D | -1.65 | D | -0.69 | D | 0.74 |
| 52 | V1471M | D | -1.3 | D | -0.42 | D | 0.72 |
| 53 | L1527H | D | -1.72 | D | -1.00 | D | 0.83 |
| 54 | L1527P | D | -1.02 | D | -1.00 | D | 0.81 |
| 55 | A1534G | D | -0.89 | D | -1.00 | D | 0.84 |
| 56 | S1535N | I | -0.34 | D | -0.54 | I | 0.50 |
| 57 | A1541P | I | -0.14 | D | -0.44 | I | 0.50 |
| 58 | I1542T | D | -2.18 | D | -1.00 | D | 0.86 |
| 59 | A1547D | D | -0.6 | I | 0.76 | I | 0.65 |
| 60 | G1586R | D | -0.02 | I | 0.17 | D | 0.70 |
| 67 | V1592D | D | -1.45 | D | -0.21 | D | 0.76 |
| 62 | Y1594S | D | -1.32 | D | -0.42 | D | 0.85 |
| 63 | I1599F | D | -1.89 | D | -1.00 | D | 0.83 |
| 64 | S1602P | I | 0.11 | D | -0.11 | D | 0.56 |
| 65 | S1602Y | I | 0.44 | I | 0.38 | I | 0.79 |
| 66 | P1603L | D | -0.14 | D | -0.35 | D | 0.78 |
| 67 | G1612D | D | -1.09 | D | -0.27 | D | 0.83 |
| 68 | A1643P | I | 0.42 | I | 1.00 | I | 0.73 |
| 69 | A1643S | I | 0.1 | I | 0.33 | I | 0.75 |
| 70 | G1644W | I | -0.01 | D | -0.75 | I | 0.54 |
| 71 | G1646S | D | -0.79 | D | -0.72 | D | 0.84 |
| 72 | G1691S | D | -1.04 | D | -0.92 | D | 0.83 |
| 73 | G1691D | D | -0.48 | D | -0.38 | D | 0.81 |
| 74 | G1691C | D | -0.93 | D | -0.60 | D | 0.82 |
| 75 | G1692S | D | -1.05 | D | -1.00 | D | 0.75 |
| 76 | P1699S | N | -1.28 | D | -1.00 | D | 0.60 |
| 77 | G1834R | D | -0.3 | I | 0.10 | I | 0.58 |
| 78 | D1870G | I | -0.26 | D | -1.00 | D | 0.55 |
| 79 | S1902R | I | 0.28 | I | 0.10 | I | 0.79 |

D-Decreasing; I-Increasing; N- Null; Variants mentioned in bold are predicted to be highly conserved from ConSurf analysis

**Table S4. Biochemical and physicochemical characterization of FLNB variants (missense) using Align GVGD server.**

| **S.No** | **Variants** | **Prediction** |
| --- | --- | --- |
| **1.** | **W148R** | **Class C65** |
| 2. | N160T | Class C55 |
| **3.** | **F161C** | **Class C65** |
| 4. | Q163P | Class C65 |
| **5.** | **W165C** | **Class C65** |
| 6. | D167E | Class C35 |
| 7. | D167G | Class C65 |
| 8. | D167V | Class C65 |
| 9. | G168S | Class C55 |
| 10. | G168V | Class C65 |
| **11.** | **G168C** | **Class C65** |
| 12. | A170P | Class C25 |
| **13.** | **L171Q** | **Class C65** |
| **14.** | **L171R** | **Class C65** |
| 15. | A173T | Class C55 |
| 16. | A173V | Class C55 |
| 17. | G181R | Class C65 |
| 18. | G181D | Class C65 |
| 19. | G181V | Class C65 |
| 20. | C183W | Class C65 |
| 21. | S188P | Class C65 |
| 22. | W189R | Class C65 |
| 23. | W189G | Class C65 |
| 24. | P191L | Class C65 |
| 25. | N197I | Class C65 |
| 26. | A201V | Class C55 |
| 27. | M202T | Class C65 |
| 28. | M202V | Class C15 |
| 29. | Q203P | Class C65 |
| 30. | A205T | Class C55 |
| 31. | W208R | Class C65 |
| 32. | G210V | Class C65 |
| 33. | I221F | Class C15 |
| 34. | E227K | Class C55 |
| 35. | S229P | Class C65 |
| 36. | L234V | Class C25 |
| 37. | S235P | Class C65 |
| 38. | G361S | Class C55 |
| 39. | G361D | Class C65 |
| 40. | G361C | Class C65 |
| 41. | G363R | Class C65 |
| 42. | G363E | Class C65 |
| 43. | R566Q | Class C35 |
| 44. | N663K | Class C65 |
| 45. | T703K | Class C65 |
| 46. | G751R | Class C65 |
| 47. | V1018M | Class C15 |
| 48. | D1157N | Class C15 |
| 49. | E1179K | Class C55 |
| 50. | R1219W | Class C65 |
| 51. | L1431R | Class C65 |
| 52. | V1471M | Class C15 |
| 53. | L1527H | Class C65 |
| 54. | L1527P | Class C65 |
| 55. | A1534G | Class C55 |
| 56. | S1535N | Class C45 |
| 57. | A1541P | Class C25 |
| 58. | I1542T | Class C65 |
| 59. | A1547D | Class C65 |
| 60. | G1586R | Class C65 |
| 67. | V1592D | Class C65 |
| 62. | Y1594S | Class C65 |
| 63. | I1599F | Class C15 |
| 64. | S1602P | Class C65 |
| 65. | S1602Y | Class C65 |
| 66. | P1603L | Class C65 |
| 67. | G1612D | Class C65 |
| 68. | A1643P | Class C25 |
| 69. | A1643S | Class C65 |
| 70. | G1644W | Class C65 |
| 71. | G1646S | Class C55 |
| 72. | G1691S | Class C55 |
| 73. | G1691D | Class C65 |
| 74. | G1691C | Class C65 |
| 75. | G1692S | Class C55 |
| 76. | P1699S | Class C65 |
| 77. | G1834R | Class C65 |
| 78. | D1870G | Class C65 |
| 79. | S1902R | Class C65 |

Variants mentioned in bold are predicted to be highly conserved from ConSurf analysis

**Table S5. The URLs are provided for the datasets and tool predictions.**

| **Dataset** | |
| --- | --- |
| **Website** | **Hyperlinks** |
| Google Scholar | https://scholar.google.com/ |
| PubMed | https://pubmed.ncbi.nlm.nih.gov/ |
| Science Direct | https://www.sciencedirect.com/ |
| PubMed Central | https://www.ncbi.nlm.nih.gov/pmc/ |
| Protein Sequence | https://www.uniprot.org |
| Protein Structure | https://www.rcsb.org/ |
| HGMD | http://www.hgmd.cf.ac.uk/ac/all.php |
| **Tool predictions** | |
| **Tools** | **Hyperlinks** |
| Align-GVGD | http:/agvgd.iarc.fr/ |
| ConSurf | http:/consurf.tau.ac.il/2016/ |
| PredictSNP | https:/loschmidt.chemi.muni.cz /predictsnp/ |
| iStable | http://predictor.nchu.edu.tw/istable/ |
| SNPeffect | http://snpeffect.switchlab.org |
| HOPE | https://www3.cmbi.umcn.nl/hope/ |
| Swiss-PDB viewer | https://spdbv.vital-it.ch/ |
